# Prevalence of urinary schistosomiasis amongst primary school children in Ikwo and Ohaukwu Communities of Ebonyi State, Nigeria

**DOI:** 10.4102/ajlm.v9i1.812

**Published:** 2020-08-24

**Authors:** Nse O. Umoh, Chimezie F. Nwamini, Nyoho J. Inyang, Anthony N. Umo, Victor U. Usanga, Amos Nworie, Michael O. Elom, Boniface N. Ukwah

**Affiliations:** 1 Department of Medical Laboratory Science, Faculty of Health Sciences and Technology, Ebonyi State University, Abakaliki, Nigeria; 2 Department of Medical Laboratory Science, Faculty of Basic Medical Sciences, Ambrose Alli University, Ekpoma, Nigeria; 3 Department of Medical Microbiology and Parasitology, College of Health Sciences, University of Uyo, Uyo, Nigeria

**Keywords:** urinary schistosomiasis, transmission, prevalence, Ebonyi State, Nigeria

## Abstract

**Background:**

Urinary schistosomiasis is a serious public health challenge in some communities of Ebonyi State, south-east Nigeria, partly resulting from a lack of adequate epidemiological data for the institution of effective control strategies.

**Objective:**

This study evaluated the prevalence and risk factors of urinary schistosomiasis in rural communities of Ebonyi State, south-east Nigeria.

**Methods:**

A total of 300 students, comprising 185 boys and 115 girls, were randomly selected for the study between July and December 2016. A questionnaire was administered to all participants to determine the risk factors for the disease in the area. Urine specimens collected from the participants were processed by sedimentation and examined microscopically for the eggs of *Schistosoma haematobium*.

**Results:**

The overall prevalence rate for urinary schistosomiasis was 8.0%. Students aged 6–10 years had the highest prevalence of infection (10.3%). The prevalence was significantly higher amongst male students (10.3%; *p* = 0.038) compared with female students (4.4%). Logistic regression analysis showed a significant association between schistosomiasis infection and freshwater contact activities (*p* = 0.007; odds ratio = 1.89; 95% confidence interval: 4.33–16.17). Contact with stream, pond, river and well water were associated with infection rates of 25%, 14%, 5.3%, and 4.4%, respectively.

**Conclusion:**

A relatively low prevalence of urinary schistosomiasis was found in the area. Participants’ socio-economic status and dependence on contaminated water sources were core modifiable risk factors. Health education and development of potable water infrastructure, amongst other interventions, would likely reduce the burden and transmission of urinary schistosomiasis in this locality.

## Introduction

Urinary schistosomiasis is a parasitic disease of the tropics and sub-tropics caused by infection of humans with the trematode (parasitic flatworm) known as *Schistosoma haematobium.* Although highly preventable, the disease ranks second only to malaria in terms of prevalence and socio-economic importance of parasitic diseases in endemic tropical and subtropical countries.^1,2,3^ Schistosomiasis remains a challenging disease of public health importance, with approximately 779 million people estimated in 2008 to be at risk globally.^4^ Worldwide, Nigeria has the highest prevalence of urinary schistosomiasis, with about 29 million cases and about 101 million people at risk of infection in 2010.^5,6,7^ The high prevalence of urinary schistosomiasis in Nigeria has been ascribed mainly to the wide distribution of *Bulinus* spp., the snail host of *S. haematobium*, and the indiscriminate passage of urine harbouring *S. haematobium* eggs by infected individuals into water lodging the snail host.^8,9^ Several factors, including poor sanitation, poverty, ignorance, limited access and availability of health facilities and social amenities, also account for the high prevalence of urinary schistosomiasis in developing countries.^10^ Human infection results when man comes into contact with water harbouring the infective stage of the parasites, the free-swimming cercariae, which have the capability of directly penetrating the water-softened, intact skin of humans who are carrying out water-related activities, such as fishing, laundry, bathing and swimming.^11,12^ The presence of the intermediate snail hosts of the parasite and increased human contact with contaminated water bodies are the key determining factors that favour the transmission of the disease. People at maximum risk are those who live in, or travel to, endemic areas and make contact with water containing the intermediate host.^8^ Children are usually prone to acquiring urinary schistosomiasis, because of a strong tendency for playing in water, which predisposes them to infection.^10^

Early detection of the parasite in infected persons is key to the control and prevention of the disease, particularly with praziquantel therapy.^13^ Parasitological diagnosis involving microscopic examination of urine for the parasite eggs is the most practical and widely-used method for detecting infected individuals;^14^ however, immunoserological diagnosis involving detection of the parasite antigens and antibodies with techniques such as an enzyme-linked immunosorbent assay have been reported to be very efficient, especially in early infections.^15^

School-age children constitute an ideal target group for investigation of urinary schistosomiasis in endemic communities, because of their known habits of poor hygiene and playing in water, which enhance the chances of infection with the parasites. The data generated from this age group have proven valuable, not only for justifying their inclusion in mass treatment programmes, but also for determining the need for such interventions.^10,16^ Urinary schistosomiasis is a serious public health challenge in some communities of Ebonyi State, south-east Nigeria.^17^ In order to provide a contemporary roadmap for establishing suitable prevention and control strategies, this study evaluated the prevalence and risk factors of the disease in some rural communities of the area.

## Methodology

### Ethical considerations

Ethical approval for this study (FHST/EC/28) was obtained from the Ethical Committee, Faculty of Health Sciences and Technology, Ebonyi State University, Abakaliki (EBSU/2016/51032).

### Study population

The participants of this cross-sectional study, conducted between July 2016 and December 2016, were selected by simple random sampling (with the aid of a Random Number Table) from amongst students of six primary schools in the Ikwo and Ohaukwu Local Government Areas (LGAs), following official authorisation by both Councils. Out of 348 pupils initially recruited from both LGAs (Ikwo, *n* = 186, Ohaukwu, *n* = 162), a total of 300 students, comprising 185 boys and 115 girls, aged between 5 and 15 years, participated in this study based on the inclusion requirements of written consent, completed questionnaire, and submission of urine specimens. A signed or thumb-printed written consent for voluntary participation of each student was obtained from a parent or guardian. Parents and guardians were informed that participants’ information would be treated with utmost confidentiality and used for the purpose of the research only before giving consent. They were also informed of the potential health and social benefits of the study. To protect the participants’ information, personal and quasi identifiers (such as occupation of the parents) were masked with numbers and letters, respectively. The sample size was determined using the formula described by Charan and Biswas^18^ for cross-sectional studies.

### Study area

This study was carried out in Ohaukwu and Ikwo LGAs of Ebonyi State, south-east Nigeria. Ohaukwu LGA is located at latitude 6°31’58.3”N and longitude 8°1’22.9”E (central part) of Ebonyi State and has an area of about 517 km^2^, with an estimated population of about 195 337, whereas Ikwo LGA is located at latitude 6°4’59”N and longitude 8°5’59”E (northern part) of Ebonyi State and has an area of about 500 km^2^, with a population of about 214 969.^19^ The two LGAs are separated from each other by a distance of about 30 km.

This area has a typical tropical climate, comprising dry and wet seasons, with an annual average rainfall of 1300 mm and an atmospheric temperature of about 30 °C; the vegetation characteristics are predominantly guinea savannah. The area is made up of several rural communities traversed by streams and rivers, which constitute the inhabitants’ major source of water for domestic use, recreation and economic activities, particularly in the absence of pipe-borne water and other social amenities. It is mostly inhabited by peasant and subsistence farmers, whose activities have an important bearing on the ecology of the area. Rice farming, mainly in swampy terrains, is the major occupation in the area.

### Administration of questionnaire and collection of samples

A questionnaire titled ‘Investigation of risk factors associated with the transmission of urinary schistiosomiasis in Ikwo and Ohaukwu Communities’ was administered verbally to each participant, parent, or guardian by the research assistants with the generous support of school tutors, who helped in communicating effectively in the local dialect. Participants’ information sought for in the questionnaire included age, residence, source(s) of water for domestic use, water contact activities, history of diseases with symptoms of haematuria, access to healthcare facilities, and the occupation and educational status of parents or guardian, amongst others.

Each participant was given a sterile dry plastic universal container with a screw lid, in which they were asked to take a terminal urine sample between 10:00 and 12:00, when the ova load is maximal.^20^ Each container was labeled with the sex, age and number of the participant as provided in the questionnaire form. Fresh urine samples collected were examined macroscopically for presence of blood (haematuria). The samples were then preserved by adding 5 mL of dilute (0.3%) carbol-fuchsin solution to each 10 mL of urine,^21^ and transported to the laboratory in an ice-pack.

### Variables definition

Categorical variables were used to assess the risks factors and prevalence of urinary schistosomiasis in this study. There were six categories, namely: LGA, sex, age, occupation, water sources, and water contact activities.

### Processing of samples

Microscopic examination of the urine samples for urinary schistosomiasis detection was based on detection of terminal-spine eggs of *S. haematobium* using a sedimentation concentration technique.^18^ Ten millilitres of each urine sample was collected from each sample container into a centrifuge tube and spun for 5 minutes at 3000 revolutions per minute to concentrate the eggs. Thereafter, the supernatant fluid was discarded into a Petri dish. A drop of the sediments was transferred to a clean and grease-free glass slide, covered with a coverslip and examined microscopically using x100 and x400 magnification for eggs of *S. haematobium*, recorded as eggs/10 mL of urine.

### Statistical analysis

Cronbach’s alpha was used to verify the reliability of the data obtained in this study. The prevalence values were calculated by finding the percentage of the factors. Associations between demographic characteristics (age, sex, and occupation) and the prevalence of infection were analysed using Pearson’s chi-square test. Multivariate logistic regression analysis was used to evaluate the geographical and behavioural risks associated with *S. haematobium* infection. The geographical variables used in the analysis model included borehole, well, pond, stream and river water sources, whilst the behavioural variables comprised swimming, fishing, washing and other domestic uses. *P*-values of less than 0.05 were considered significant, and 95% confidence intervals were used to locate outliers.

## Results

### Prevalence of urinary schistosomiasis in Ohaukwu and Ikwo

*Schistosoma haematobium* infection was detected in 24 (8.0%) of the 300 students examined. The prevalence of infection was 10.0% (*n* = 150) in Ikwo LGA and 6.0% (*n* = 150) in Ohaukwu LGA. The difference between the prevalence of infection in Ohaukwu and Ikwo LGAs was not statistically significant (*p* = 0.24; Table 1).

**TABLE 1:** Prevalence of urinary schistosomiasis in Ohaukwu and Ikwo, Nigeria, December 2016.

LGA†	No. examined	No. infected	Prevalence (%)	p*
Ikwo	150	15	10.0	0.24
Ohaukwu	150	9	6.0	-

**Total**	**300**	**24**	**8.0**	**-**

LGA, local government area; No., number; *p*, probability.

* , Pearson’s chi-square test for Ikwo and Ohaukwu LGAs.

† , Locations: Ohaukwu LGA – Amawule, Umuezeaka, and Ndiagu communities; Ikwo LGA – Abina, Ebianya Noyo, and Obeagu Omege communities.

### Demographic distribution of students with *Schistosoma haematobium* infection

The highest prevalence of infection by age was 9.4% (*n* = 160) amongst students aged 6–10 years. Students aged 11–15 years had a lower prevalence rate of 7.0% (*n* = 72), followed by those aged 1–5 years (*n* = 50; 6.0%), whereas students aged more than 15 years had the lowest (*n* = 18; 5.5%). The association between prevalence of infection and age of the participants was not statistically significant (*p* = 0.84; Table 2).

**TABLE 2:** Demographic distribution of subjects with *Schistosoma haematobium* infection in Ohaukwu and Ikwo, Nigeria, December 2016.

Demographic features	No. examined	No. infected	Prevalence (%)	p*
**Age**				0.84
≤ 5	50	3	6.0	
6–10	160	15	9.4	
11–15	72	5	7.0	
> 15	18	1	5.5	
**Gender**				0.038**
Male	185	19	10.3	
Female	115	5	4.3	
**Occupation**				0.23
Farming	128	15	11.7	
Fishing	26	1	3.8	
Trading	85	6	7.1	
Public service	61	2	3.3	

No., number; *p*, probability.

* , Pearson’s chi-square analysis for age groups (≤ 5, 6–10, 11–15, > 15), gender (male, female) and occupation of parents (farming, fishing, trading, public service);

** , Statistically significant (*p* < 0.05).

Of the 185 male students, 19 (10.3%) were infected with *S. haematobium* compared with 5 (4.3%) of the 115 female students. The impact of sex on the prevalence of infection was statistically significant (*p* = 0.038; Table 2).

The highest prevalence of infection by occupation (*n* = 128; 11.7%) was found amongst students whose parents or guardians were farmers, whilst children of public servants had the least (*n* = 61; 3.2%) (Table 2). A prevalence of 7.0% (*n* = 85) was found amongst children whose parents or guardians were traders, and 3.8% (*n* = 26) where the parents or guardians were fishermen. The relationship between the prevalence of infection and the occupation of parents was not statistically significant (*p* = 0.23; Table 2).

### Association between sources of water/contact and *Schistosoma haematobium* infection

The highest rate of infection (*n* = 48; 25%) was found amongst students who utilised stream water mainly for domestic purposes. Infection rates of 14.0% (*n* = 50) were found amongst those that used pond water, 5.3% (*n* = 57) for river water, and 4.4% (*n* = 45) for well water. There was no case of infection amongst students who utilised water from borehole facilities; the odds of infection were about three times higher with participants who utilised freshwater sources within the locality compared with those who used borehole water, but this association was not statistically significant (*p* = 0.55; odds ratio = 2.77; 95% confidence interval: 7.23–22.77; Table 3).

**TABLE 3:** Association between sources of water and *Schistosoma haematobium* infection (*n* = 300), Nigeria, December 2016.

Water sources	No. examined	% infected	OR† (CI of mean)	p*
Borehole	100	0.0	2.77 (7.23–22.77)	0.55**
Well	45	4.4		
Pond	50	14.0		
Stream	48	25.0		
River	57	5.3		

*n*, sample size; No., number; OR, odds ratio; CI, confidence interval; *p*, probability.

* , Odds ratio for freshwater (well, pond, stream and river) and borehole water sources.

** , Statistically insignificant (*p* > 0.05).

† , Logistic regression analysis; model adjusted for borehole, well, pond, stream and river water sources.

A high infection rate of 10.5% (*n* = 95) was found amongst participants who engaged in swimming, compared with other activities that involved contact with freshwater sources (Table 4). Multivariate analysis showed a statistically significant association between participants’ water contact activities and the prevalence of infection (*p* = 0.007; odds ratio = 1.89; 95% confidence interval: 4.33–16.17).

**TABLE 4:** Distribution of *Schistosoma haematobium* infection by freshwater contact activities (*n* = 270), Nigeria, December 2016.

Activities	Participation	% infected	OR† (CI of mean)	P*
Swimming	95	10.5	1.89 (4.33–16.17)*	0.007**
Fishing	25	8.0		
Washing	70	8.5		
Domestic uses	80	5.3		

*n*, sample size; No., number; OR, odds ratio; CI, confidence interval; *p*, probability.

* , Logistic regression analysis; model adjusted for swimming, fishing, washing and domestic uses.

** , Statistically significant (*p* < 0.05).

† , Odds ratio for swimming, fishing, washing and domestic uses.

## Discussion

Urinary schistosomiasis is reported to be endemic in virtually all rural regions of Nigeria, because of a widespread occurrence of ecological and socio-economic factors associated with the disease.^8,9,22,23^ This study confirmed the existence of urinary schistosomiasis in Ebonyi State, south-east Nigeria with a prevalence of 8.0% in the study area. This prevalence rate was low compared with previous studies in the area, which reported higher rates, 49.7% for Ohaukwu LGA and 11.0% for Onicha LGA.^17^ The low prevalence rate of the disease found in the present study could, firstly, be attributed to the impact of a preventive praziquantel-treatment programme, initiated by the World Health Organization in 2014, for school-aged children and special risk groups in the area.^24^ Secondly, development of private borehole water facilities often for commercial purposes is trending in some communities of this locality. Although not accessible or affordable to a large segment of this rural population for patronage, access to this source of water by some residents, mainly for domestic uses, may have drastically reduced the risk of contact with parasite-infested water sources. This study found a higher prevalence of infection in communities without such facilities. Based on such a finding, it would be rather surprising that the disease prevalence was lower in Ohaukwu than Ikwo LGA, which has a few communities in close proximity to infrastructural and healthcare facilities in the State capital city, Abakaliki. Access to such amenities by the inhabitants of these communities should essentially impact positively on their living conditions, with a likelihood of reducing the prevalence level of the disease. This was, however, not the case, perhaps because of a combination of factors, including poor perception of the disease transmission dynamics, and a high probability of occupation-related infections in these agrarian communities, where parents often go to rice farms in the company of their children. Nevertheless, the association between parents’ occupation and the prevalence of urinary schistosomiasis in this study was not statistically significant (*p* = 0.235). Previous studies in some parts of Nigeria had reported high prevalence rates of 48.8% (Bauchi State), 44% (Adamawa State), 55.7% (Cross River State), and 21.5% (Ebonyi State), which were associated mainly with the predominant occupation of the indigenes, such as fishing and farming.^25,26,27,28,29^

In concordance with reports from many parts of Nigeria,^28,30,31^ this study found the influence of sex to be an important epidemiological factor in the transmission of urinary schistosomiasis, with a significantly higher prevalence of infection amongst male students compared with female students (*p* = 0.038). This agrees with the finding of Okoli and Odaibo^32^ in a previous study that attributed higher infection rates of urinary schistosomasis amongst school boys in Ibadanto to a greater involvement in outdoor activities, such as swimming, washing, paddling of canoes, and irrigation. However, persons who have greater contact with the snail breeding loci are more likely to acquire the infection, regardless of their sex.^28^ Most students in primary school classes are in the 6–10-year age bracket, and therefore it was not surprising that this age group had the highest prevalence of infection, perhaps on account of greater involvement in water-related activities compared with other groups, and not necessarily due to increased vulnerability. Children are generally known to be vulnerable to urinary schistosomiasis, because of their strong tendency to play in water, which predisposes them to the infection.^10^

### Limitations

Regardless of the age or sex of students, contact with freshwater sources was a crucial factor for disease transmission, based on the pattern of infection found in this study. The prevalence of the disease was particularly high amongst children who utilised stream water for domestic and other uses, compared with other sources of water, including ponds and river. No case of infection was found amongst students who had unlimited access to borehole water. This finding is consistent with the fact that *S. haematobium* infection occurs only where there is contact between the population and freshwater sources harbouring the snail vector as well as the infective stage of the parasite.^30^ Thus, this study may have been considerably limited by an inability to examine the water sources for the snail host, and quantify the participants’ water contact activities. Nonetheless, a significant association was found between infection and students’ water contact activities. The zero-prevalence rate of the disease found amongst students who utilised borehole water facilities in this locality may underscore the development of infrastructure in rural communities as an important control approach for urinary schistosomiasis.

### Conclusion

This study reports a low prevalence of 8.0% for urinary schistosomiasis amongst children in the study area, with a likelihood of further expansion in the disease rate and foci, if appropriate control measures are not initiated urgently by the local health authorities. Transmission of the disease in these communities is enhanced mainly by residents’ dependence on unsafe sources of water for domestic use, as well as behavioural and socio-economic tendencies that promote risky contacts with potentially parasite-infested water bodies. Continued disease surveillance and selective praziquantel chemotherapy for infected persons, health education of the residents for improved perception of the behavioural and socio-economic activities associated with the disease, and development of water sources would likely improve the living conditions of this rural population with a consequent reduction in the burden and transmission of the disease. Future studies on urinary schistosomiasis in these communities should include specific analysis of the water bodies, including malacological evaluation, as well as quantification of the water contact activities of the participants.
